# Noninvasive technique to evaluate the muscle fiber characteristics using q-space imaging

**DOI:** 10.1371/journal.pone.0214805

**Published:** 2019-04-04

**Authors:** Junichi Hata, Daisuke Nakashima, Osahiko Tsuji, Kanehiro Fujiyoshi, Kaori Yasutake, Yasushi Sera, Yuji Komaki, Keigo Hikishima, Takeo Nagura, Morio Matsumoto, Hideyuki Okano, Masaya Nakamura

**Affiliations:** 1 Central Institute for Experimental Animals, Kawasaki, Kanagawa, Japan; 2 RIKEN Brain Science Institute, Wako, Saitama, Japan; 3 Department of Physiology, Keio University School of Medicine, Shinjuku, Tokyo, Japan; 4 Department of Orthopaedic Surgery, Keio University School of Medicine, Shinjuku, Tokyo, Japan; 5 Department of Orthopaedic Surgery, Murayama Medical Center, Murayama, Tokyo, Japan; 6 Institute for Integrated Sports Medicine, Keio University School of Medicine, Shinjuku, Tokyo, Japan; 7 Okinawa Institute of Science and Technology Graduate University, Kunigami, Okinawa, Japan; 8 Department of Clinical biomechanics, Keio University School of Medicine, Shinjuku, Tokyo, Japan; University at Buffalo, UNITED STATES

## Abstract

**Background:**

Skeletal muscles include fast and slow muscle fibers. The tibialis anterior muscle (TA) is mainly composed of fast muscle fibers, whereas the soleus muscle (SOL) is mainly composed of slow muscle fibers. However, a noninvasive approach for appropriately investigating the characteristics of muscles is not available. Monitoring of skeletal muscle characteristics can help in the evaluation of the effects of strength training and diseases on skeletal muscles.

**Purpose:**

The present study aimed to determine whether q-space imaging can distinguish between TA and SOL in *in vivo* mice.

**Methods:**

*In vivo* magnetic resonance imaging of the right calves of mice (n = 8) was performed using a 7-Tesla magnetic resonance imaging system with a cryogenic probe. TA and SOL were assessed. q-space imaging was performed with a field of view of 10 mm × 10 mm, matrix of 48 × 48, and section thickness of 1000 μm. There were ten b-values ranging from 0 to 4244 s/mm^2^, and each b-value had diffusion encoding in three directions. Magnetic resonance imaging findings were compared with immunohistological findings.

**Results:**

Full width at half maximum and Kurtosis maps of q-space imaging showed signal intensities consistent with immunohistological findings for both fast (myosin heavy chain II) and slow (myosin heavy chain I) muscle fibers. With regard to quantification, both full width at half maximum and Kurtosis could represent the immunohistological findings that the cell diameter of TA was larger than that of SOL (*P* < 0.01).

**Conclusion:**

q-space imaging could clearly differentiate TA from SOL using differences in cell diameters. This technique is a promising method to noninvasively estimate the fiber type ratio in skeletal muscles, and it can be further developed as an indicator of muscle characteristics.

## Introduction

Skeletal muscles are heterogeneous tissues that include slow and fast muscle fibers [1]. The types of muscle fibers are based on the myosin heavy chain (MHC) isoform compartment and include one slow type (MHC I) and multiple fast types (MHC IIa, MHC IIb, MHC IId, and MHC IIx) [1]. The tibialis anterior muscle (TA) and soleus muscle (SOL) have been widely used in physiological and pathological studies involving animals and humans [2]. TA is mainly composed of fast muscle fibers, whereas SOL is mainly composed of slow muscle fibers [3]. The distribution of fast and slow muscle fibers is influenced by multiple factors, including neuromuscular activity and passive mechanical loading [4, 5], aging [6, 7], and hormonal balance [8]. These changes are associated with MHC isoform compartment expression [9].

In order to assess the changes in muscle fibers for monitoring the effects of strength training, it is important to assess the effects of waste syndrome involving aging and the effects of treatment. Although biopsy may be used to assess the characteristics of muscles, no appropriate noninvasive method exists.

In recent years, studies using diffusion-weighted imaging (DWI) for assessing muscles, especially diffusion tensor imaging (DTI) [10–14], have been reported. Various factors, such as sex [11], muscle macro-morphological structure (parallel muscle or bipennate muscle) [11], and age [15], can affect DTI values. Conversely, this approach is not sensitive enough to detect slight differences between slow and fast muscle fibers, which have differences in cell size [16]. Additionally, conventional DWI, including DTI, is based on the theory that water molecules follow Gaussian distribution. However, living tissue, including muscle fibers, have a restricted environment that obstructs the distribution of water molecules, and therefore, the distribution of water in muscle fibers is far from Gaussian distribution. Diffusion signal is affected by numerous factors, including water restriction, water exchange, and variations in tissue compartment size. Hence, different approaches that are not based on Gaussian distribution are required to address all the issues affecting the signal in DWI.

q-space imaging (qsi) is a quantitative DWI approach that allows the detection of minor changes in the microstructure of environments wherein the movement of water is restricted [17, 18]. In contrast to conventional DWI, qsi does not assume Gaussian distribution for the underlying probability density function (PDF) of water molecule diffusion [19–21]. Studies have shown that qsi reflects the microstructure of tissues *in vivo* [19–21]. qsi can provide quantitative diffusion values, including full width at half maximum (FWHM; μm), Kurtosis [arbitrary unit (a.u.)], and probability at zero displacement (a.u.), and these are obtained from the shape of PDF [22, 23]. Thus, more precise microstructural information can be obtained with qsi than with DTI *in vivo*. qsi has been reported to be useful for the diagnosis and therapeutic evaluation of multiple sclerosis [19, 23–26] and the diagnosis of carcinoma in *ex vivo* studies [27, 28]. In the field of muscle research, Erik et al. reported on the validity of qsi for analyzing muscle fiber direction rather than muscle cell structure [29]. There is no report on the utility of qsi for evaluating the characteristics of muscle cell structure.

In this study, we aimed to determine whether qsi can distinguish between TA and SOL in an *in vivo* mouse model. To the best of our knowledge, this is the first study to examine the feasibility of qsi for evaluating muscle quality *in vivo*.

## Materials and methods

The study included eight female C57BL/6J mice aged 8–16 weeks. The mice were born and kept under specific pathogen-free conditions and were cared for in accordance with the guidelines of the Central Institute for Experimental Animals. *In vivo* magnetic resonance imaging (MRI) of the right calf was performed with the mice in the prone position on an imaging stretcher under anesthetization with 2.0% isoflurane (Abbott Laboratories, Abbott Park, IL, USA) in an oxygen and air mixture. After MRI, all mice were sacrificed under anesthetization with 5.0% isoflurane in an oxygen and air mixture. The right calf was dissected and quickly frozen in isopentane (26404–75; Nacalai Tesque, Kyoto, Japan). The harvested right calves were subjected to frozen sectioning according to a previous report [30], and 10-μm-thick sections were obtained. This study was approved by the institutional review board of the Central Institute for Experimental Animals (approval number, 15058A).

### MRI protocol

**Fig 1:**
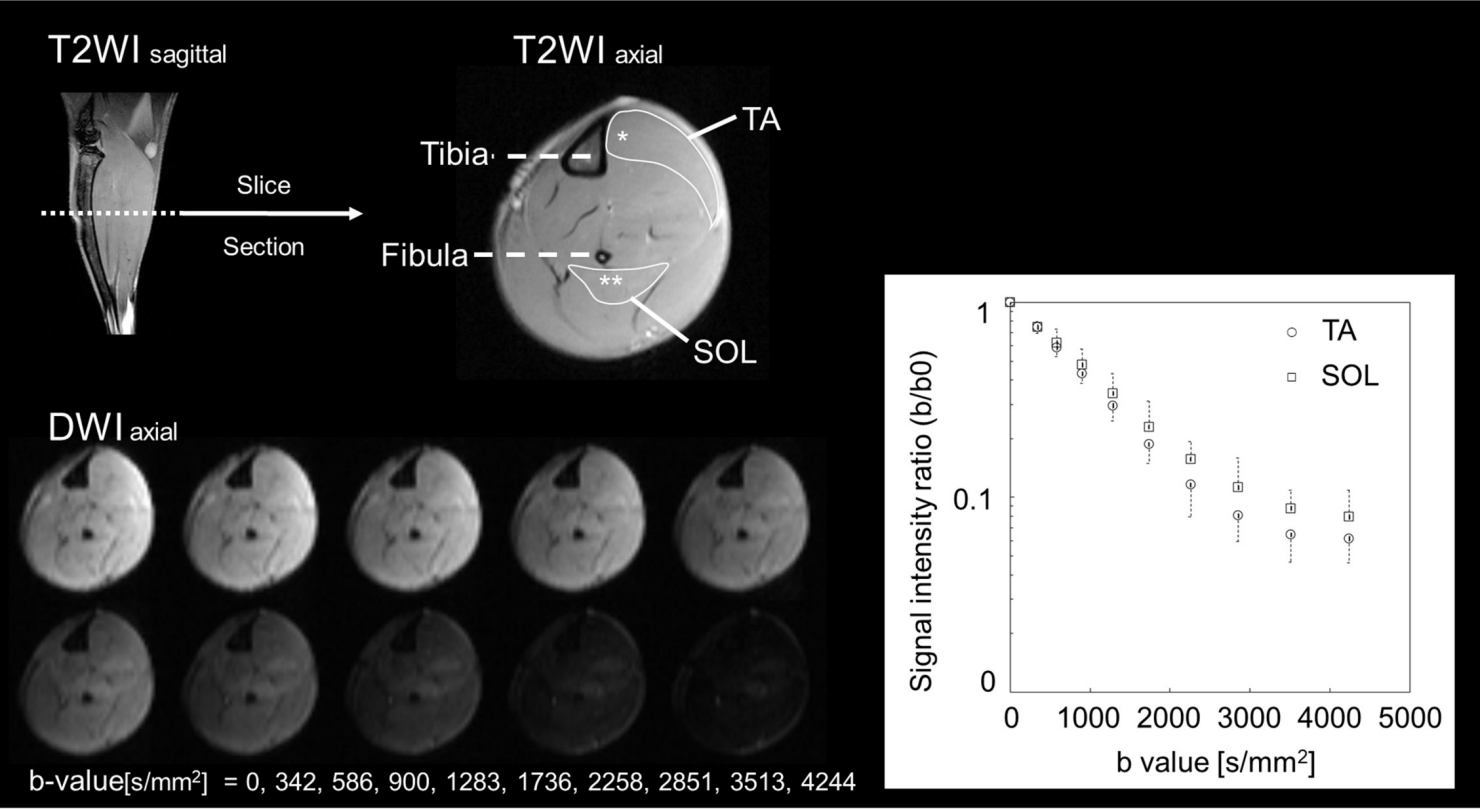
Magnetic resonance imaging (MRI) diffusion raw data. Axial and sagittal T2-weighted MRI scans were performed as reference scans for muscle identification and measurement. The region with the maximum circumferential diameter in the calf is confirmed. Region of interests of tibialis anterior muscle (TA) and soleus muscle (SOL) for MRI analysis were shown in T2-weighted axial image. We identified the muscles in contact with the tibia and fibula as markers and obtained the histologies from the vicinity of these bones. Axial diffusion-weighted image with the largest calf circumference was used. TA: tibialis anterior muscle, SOL: soleus muscle, *: biopsy site of TA, **: biopsy site of SOL.

MRI was performed using a 7.0-Tesla system (BioSpec 70/16; Bruker BioSpin, Ettlingen, Germany) with a cryogenic 2-channel surface probe (Bruker BioSpin AG, Fällanden, Switzerland) to improve sensitivity [31, 32] and to obtain high-resolution images (Fig 1). Axial and sagittal T2-weighted imaging (T2WI) scans were performed as reference scans for muscle identification and measurement. Subsequently, qsi was performed.

The imaging parameters for axial T2WI were as follows: rapid acquisition with relaxation enhancement; repetition time (TR), 3000 ms; echo time (TE), 12 ms; mean, 4; field of view (FOV), 10 × 10 mm^2^; matrix size, 200 × 200; resolution, 50 × 50 μm^2^; slice thickness, 600 μm; and imaging time, 3 min 42 s.

The imaging parameters for qsi using diffusion-weighted pulsed-gradient stimulated-echo (PGSTE) planner imaging were as follows: TR, 3000 ms; TE, 17.97 ms; mean, 2; FOV, 10 × 10 mm^2^; matrix size, 48 × 48; resolution, 208 × 208 μm^2^; slice thickness, 1000 μm; acquisition time, 19 min 36 s. There were ten b-values (0, 342, 586, 900, 1283, 1736, 2258, 2851, 3513, and 4244 s/mm^2^), and each b-value had diffusion encoding in three directions ([x, y, z] = [1, 0, 0], [−1, $\sqrt{3}/2$, 0], [−1, $-\sqrt{3}/2$, 0]). The corresponding q-values for the ten b-values were 0, 77.3, 109.3, 133.8, 154.5, 172.8, 189.2, 204.4, 218.5, and 231.8 cm^−1^, respectively. The gradient length (δ) and time between the two leading edges of the diffusion gradient (Δ) were 3.6 and 201.2 ms, respectively. Data from the three directions were acquired separately and then averaged. Thus, a cross section perpendicular to the direction of the muscle fiber was measured and then averaged with regard to the three directions. The signal-to-noise ratio (SNR) of b0 images ranged from 31.2 to 48.3, indicating that the signal levels were sufficient in relation to the noise levels. Cohen and Assaf reported that there is no change in qsi parameters when the SNR is 20 or more [33]. We considered that the SNR in this study was sufficient for performing qsi.

### Imaging analysis

qsi analyses were performed using an in-house program (developed in C++; Embarcadero Technologies, Inc., Austin, TX, USA). The non-Gaussian PDF of water diffusion was obtained by performing Fourier transformation of the data on the basis of the Stejskal–Tanner diffusion preparation [34]. Detailed new diffusion values and their calculation procedures were as previously described [19, 20, 22]. Briefly, by performing Fourier transformation, the following formula was obtained:

$$P(R,\Delta )=1/((\sqrt{4\pi D\Delta })*exp(-R2/4D\Delta ))$$ (1)

The PDF [P(R, Δ)] was accordingly calculated. If the object to be measured includes one element, the PDF is considered to show Gaussian distribution. In contrast, if the object includes several elements, the PDF is considered to show non-Gaussian distribution, reflecting the complexity of the measured object. The important theory in qsi is that Fourier transformation of the signal decay with respect to the b-value provides the PDF for diffusion using multiple q-values [19]. In this case, the q-value, which represents the horizontal axis, was defined by the following formula:

q=γδG/2π (2)

The FWHM (μm) and Kurtosis (a.u.) maps were obtained. The shape of the PDF can be characterized by FWHM. Kurtosis values were calculated using the following formula:

$$Kurtosis=\frac{1}{N}\sum (\frac{x_{i}-\bar{x}}{SD}{)}^{4}-3$$ (3)

where N is the number of data points (i.e., number of b-value steps used), x is the probability value (a.u.) obtained from the PDF, and SD is the standard deviation of the x values.

### Region of interest

In the present study, the TA and SOL area were identified on T2WI. The axial image with the largest calf circumference was used. Each qsi value was measured by two experts (J.H. and D.N.; 8 and 10 years of experience, respectively) with consensus. Structures outside the muscle were carefully avoided. To assess the reproducibility of the calculations, blinded analyses were performed, and they involved independent triplicate analyses on different days. Reproducibility was quantified using the intraclass correlation coefficient (ICC) for absolute agreement. The ICC values were interpreted as follows: 0.81–1.0, substantial agreement; 0.61–0.80, moderate agreement; 0.41–0.60, fair agreement; 0.11–0.40, slight agreement; and 0.00–0.10, virtually no agreement [35]. An ICC value >0.81 was considered to represent good agreement.

The imaging values were measured using the software imageJ 1.48v (available at: rsbweb.nih.gov/ij/; National Institutes of Health, Bethesda, MD, USA). Color mapping images were created using the software Mango, version 4.0.1 (available at: rii.uthscsa.edu/mango).

### Immunohistochemistry

The frozen sections of mice calves were post-fixed with methanol for 10 min at −30°C. Immunohistochemistry was performed according to standard procedures [30]. In order to define the histology site from the same location as the location of region of interest in MRI analysis, we identified the muscles in contact with the tibia and fibula as markers and performed biopsy from the vicinity of these bones. The following mouse monoclonal antibodies were used as primary antibodies: BA-D5 (isotype IgG2b; Developmental Studies Hybridoma Bank [DSHB]; dshb.biology.uiowa.edu) for MHC I, SC-71 (isotype IgG1; DSHB) for MHC IIa, and BF-F3 (isotype IgM; DSHB) for MHC IIb [36]. For each section, triple staining (BA-D5, pink; SC-71, green; and BF-F3, red) was performed, and it involved incubation with appropriate fluorescent-conjugated secondary antibodies [Alexa Fluor 647 for IgG2b, Alexa Fluor 488 for IgG1, and Alexa Fluor 555 for IgM (Thermo Fisher Scientific Inc., Waltham, MA, USA)]. Nuclei were stained with Hoechst 33342 (Sigma-Aldrich Inc., St. Louis, MO, USA).

The antibody combination used did not consider MHC IId/x, and it remained unlabeled according to a previous report [37]. All stained specimens were viewed under a confocal laser scanning microscopy (LSM700; Carl Zeiss Microscopy, NY, USA).

The frequencies of BA-D5-positive cells, SC-71-positive cells, BF-F3-positive cells, and unlabeled cells were measured for each muscle. In addition, the diameter of each cell was measured for TA and SOL. The imaging values were measured using the software imageJ 1.48v at a magnification of 200×. Three individual areas were selected at random, and the mean frequency and diameter were calculated. All the sections were carefully surveyed to identify regions free of artifacts. Overall, 754 individual muscle fibers could be accurately characterized in all sections.

### Statistical analysis

Data are presented as the mean, standard deviation (SD), and minimum and maximum. Student’s *t*-test was used to evaluate the relationship between the TA and SOL with regard to the frequency of each cell type on immunohistology, the histological cell diameter, and each qsi parameter (FWHM [μm] and Kurtosis [a.u.]). Additionally, Student’s *t*-test was performed to evaluate the relationship among the MHC types of muscle cells. All analyses were performed using SPSS statistical software, version 24 (IBM Corp., Armonk, NY, USA). The significance level for all tests was set at a *P*-value <0.05.

## Results

### Immunohistochemistry

**Fig 2:**
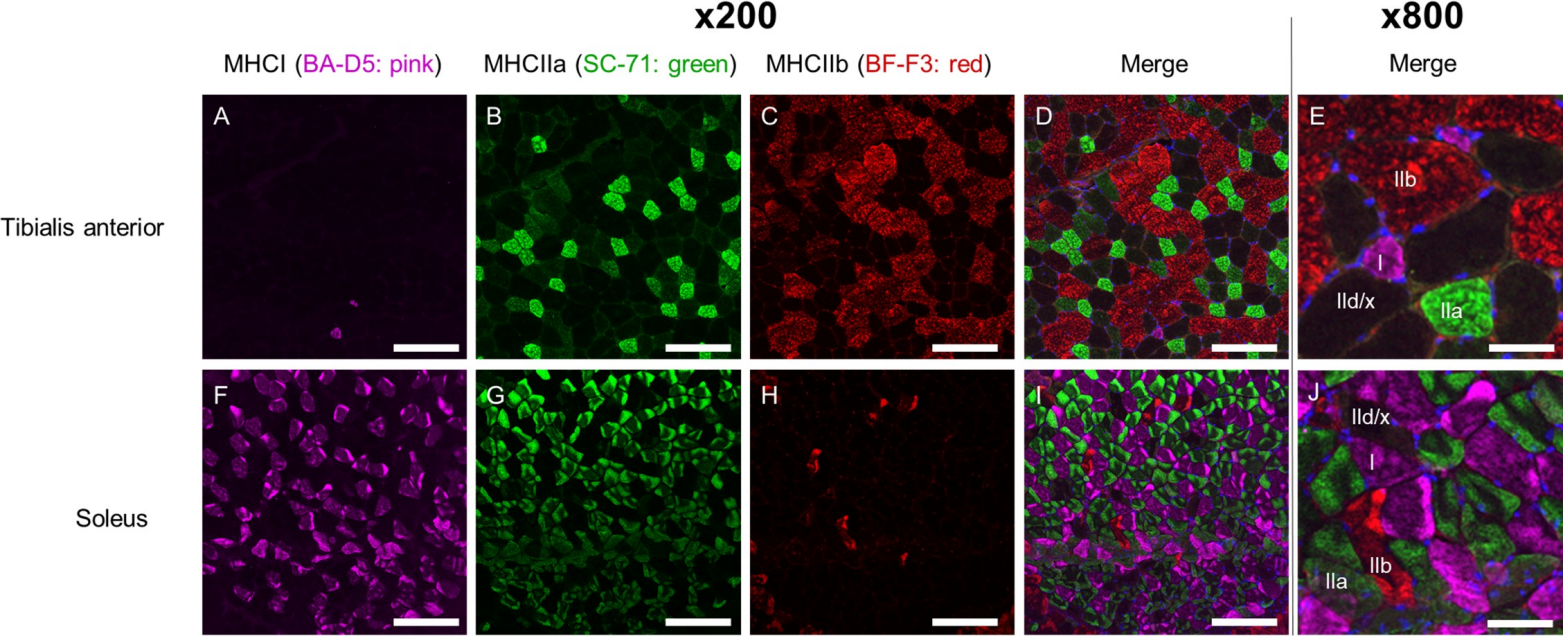
**Immunohistological analysis of the tibialis anterior muscle (A-E) and soleus muscle (F-J).** BA-D5-positive cells, pink; SC-71-positive cells, green; and BF-F3-positive cells, red (A-D and F-J: 200×, scale bar = 100 μm; E and J: 800×, scale bar = 25 μm).

Representative images of immunohistochemical staining are presented in Fig 2. MHC I cells (slow muscle fiber) appeared to be more common in SOL than in the (Fig 2A and 2F), and this finding is similar to a previous finding [38].

**Fig 3:**
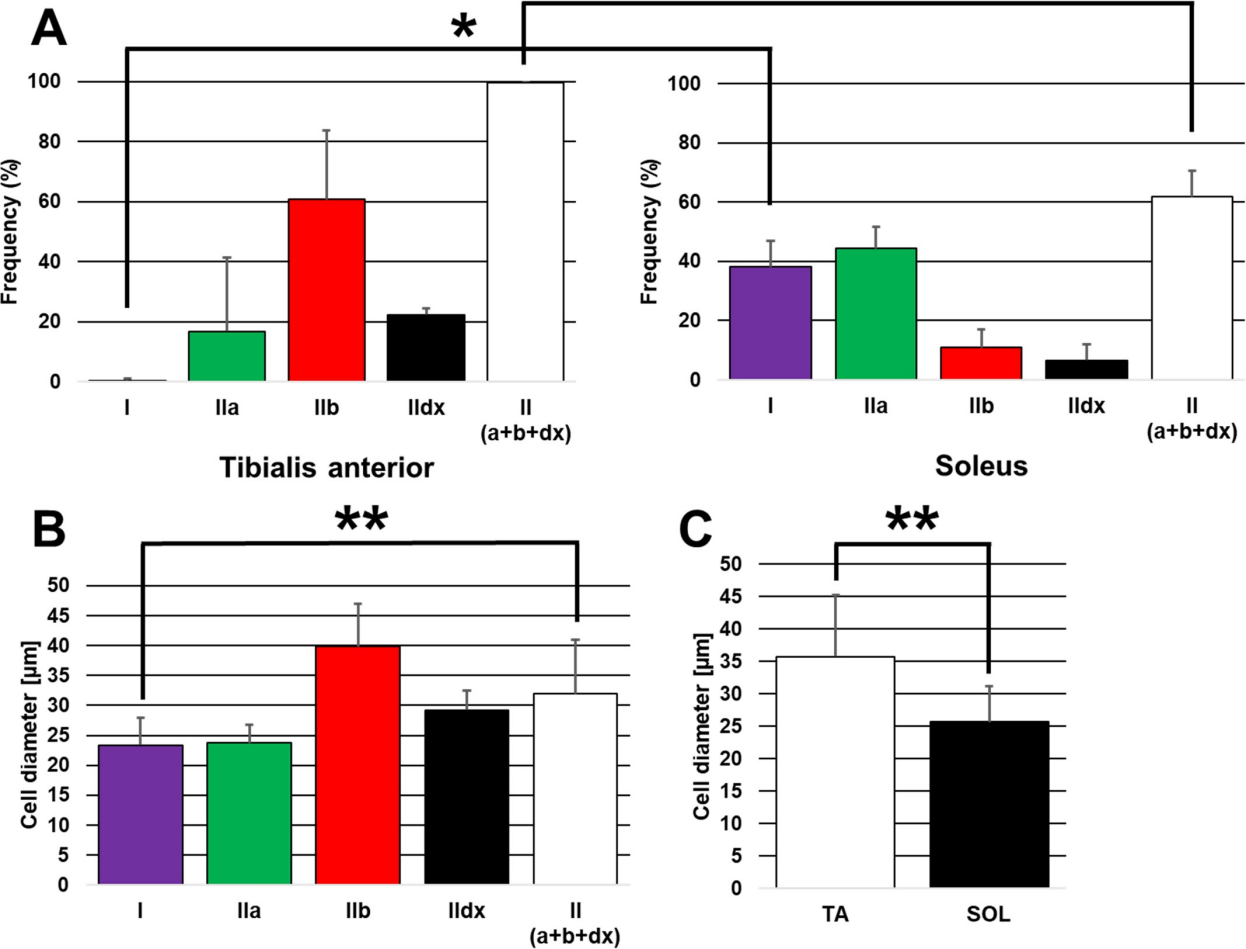
Quantification of the immunohistological findings. A: Frequency according to MHC type in each muscle. The frequency of MHC type I cells was significantly higher in the soleus muscle (SOL) than in the tibialis anterior muscle (TA), whereas the frequency of MHC type II cells was significantly higher in TA than in SOL. B: Cell diameter according to MHC type in TA and SOL. The mean diameter of MHC type I cells was significantly lower than that of MHC type II cells of TA and SOL. C: General cell diameter in each muscle (TA and SOL). The mean cell diameter in the TA was significantly higher than that in the SOL. The end of the whisker represents standard deviation. *P < 0.05; **P < 0.01.

Quantification of the immunohistological findings is presented in Fig 3, and summaries of the immunohistological results are presented in Table 1 and Table 2. The frequency of MHC I cells (slow muscle fiber) was significantly higher in SOL than in the (*P* < 0.05) (Fig 3A). Conversely, the frequency of MHC II cells (type IIa, b, and d/x; fast muscle fiber) was significantly higher in TA than in SOL (*P* < 0.05) (Fig 3A). The mean diameter of MHC I cells (slow muscle fiber) was significantly lower than that of MHC II cells (type IIa, b, and d/x; fast muscle fiber) (*P* < 0.05) (Fig 3B). Thus, the mean cell diameter of TA was significantly higher than that of SOL (*P* < 0.05) (Fig 3C and Table 3).

**Table 1:** Frequency of each cell according to the MHC type.

Parameter	MHC type	Unit	Tibialis anterior		Soleus	
			Mean (SD)	Min–Max	Mean (SD)	Min–Max
Frequency	I	(%)	0.338 (0.586)	0–1.02	38.2 (8.79)	32.0–48.2
	IIa		16.7 (24.7)	1.53–45.2	44.3 (7.22)	37.3–51.8
	IIb		60.7 (23.2)	34.0–75.6	10.9 (5.98)	7.46–47.3
	IIdx		22.3 (2.25)	19.8–24.2	6.59 (5.45)	0.33–11.0
	II(a + b + dx)		99.7 (0.586)	99.0–100	61.8 (8.79)	59.7–97.2

**Table 2:** Diameter of each cell according to the MHC type.

Parameter	MHC type	Unit	Mean (SD)	Min–Max
Cell diameter	I	(μm)	23.4 (4.51)	6.86–31.7
	IIa		23.7 (3.05)	16.6–31.6
	IIb		39.8 (7.12)	23.4–67.1
	IIdx		29.2 (3.18)	22.9–38.3
	II(a + b + dx)		31.9 (8.97)	16.6–67.1

**Table 3:** Summary of cell sizes of the tibialis anterior muscle (TA) and soleus muscle (SOL) according to immunohistology and q-space imaging parameters.

Parameter	Muscle	Unit	Mean (SD)	Min–Max
Cell diameter	TA	(μm)	35.7 (9.47)	14.0–67.1
	SOL		25.6 (5.47)	6.86–42.3
FWHM	TA	(μm)	29.2 (2.50)	26.9–34.8
	SOL		23.7 (1.64)	20.8–26.4
Kurtosis	TA	(a.u.)	1.21 (0.0637)	1.10–1.28
	SOL		1.50 (0.0212)	1.46–1.53

FWHM, full width at half maximum

### qsi

To assess the reproducibility of qsi calculation, blinded assessment (drawing region of interests) involving independent triplicate analyses was performed. The ICC for intraobserver reproducibility was 0.912, indicating good reproducibility among the three analyses, and the ICC for interobserver reproducibility was 0.928, indicating good reproducibility among the observers.

**Fig 4:**
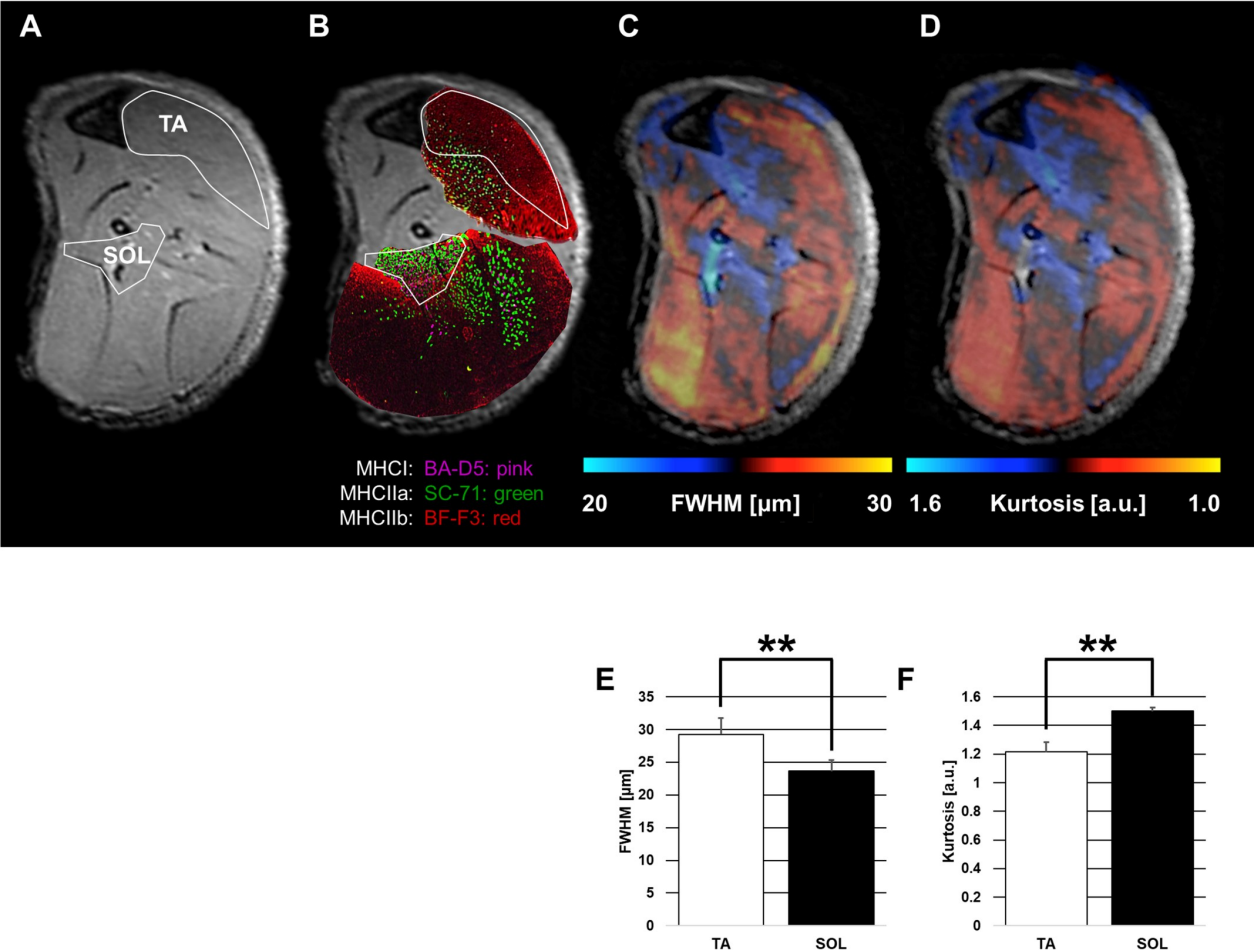
Comparison of the immunohistological findings and qsi findings in the tibialis anterior muscle (TA; fast muscle fiber) and soleus muscle (SOL; slow muscle fiber). A: Regions of interest on axial T2-weighted imaging (T2WI; low power field) with the largest calf circumference as reference. B: Immunohistological findings (low power field, ×1) on axial T2WI. The legions of TA and SOL at Fig 4A are shown. TA is mainly composed of BF-F3-positive cells and SOL is composed of BA-D5-positive cells and SC-71-positive cells. BA-D5-positive cells, pink; SC-71-positive cells, green; and BF-F3-positive cells, red C: Full width at half maximum (FWHM) map on axial T2WI. The bright blue (low FWHM) lesion is consistent only at the SOL lesion. D: Kurtosis map on axial T2WI. The white (high Kurtosis) lesion is consistent only at the SOL lesion. E: FWHM findings for each muscle (TA and SOL). F: Kurtosis findings for each muscle (TA and SOL). The end of the whisker represents standard deviation. **P < 0.01 Student’s *t*-test was used to evaluate the relationship between the TA (fast muscle fiber) and SOL (slow muscle fiber) with regard to qsi parameters (FWHM and Kurtosis).

FWHM (Fig 4C) and Kurtosis (Fig 4D) maps showed the signal intensities consistent with immunohistological findings (Fig 4B). The areas of BA-D5-positive cells (type I fiber; pink) and SC-71-positive cells (type IIa fiber; green) in Fig 4B (SOL area) match the distributions for FWHM (under 25 μm) in Fig 4C and Kurtosis (over 1.3 a.u.) in Fig 4D. In addition, the area of BF-F3-positive cells (type IIb fiber; red) in Fig 4B (including the TA area) matches the distributions for FWHM (over 25 μm) in Fig 4C and Kurtosis (under 1.3 a.u.) in Fig 4D. In qsi, a larger FWHM or smaller Kurtosis value indicates a larger cell diameter. This is consistent with the fact that BF-F3-positive cells have the largest diameter, followed by SC-71-positive cells and BA-D5-positive cells. The findings of both qsi parameters (FWHM; Fig 4E and Table 3 and Kurtosis; Fig 4F and Table 3) were consistent with the immunohistological finding that the cell diameter of the TA was higher than that of the SOL quantitatively (*P* < 0.01) (Fig 3C and Table 3).

## Discussion

In this study, by focusing on cell size, a significant difference was noted between TA (mainly containing fast muscle fibers) and SOL (mainly containing slow muscle fibers) on qsi. Our findings indicate that high FWHM and low Kurtosis values are characteristic of TA, whereas low FWHM and high Kurtosis values are characteristic of SOL.

A previous histological study reported that among MHC I, MHC IIa, MHC IIb, and MHC IId/x cells, MHCIIb cells were the largest, followed by MHC IId/x, MHC IIa, and MHC I cells [16]. Additionally, MHC I cells were predominant in SOL, whereas MHC II cells were predominant in TA [39]. These findings indicate that the fast muscle fiber-dominant TA generally has a large cell diameter, whereas the slow muscle fiber-dominant SOL generally has a small cell diameter. Our immunohistological results are consistent with these previous findings.

In MRI studies, it was not possible to represent the histological cell distribution on T2WI. Conversely, qsi produced contrast images that were consistent with histological cell distribution qualitatively. In quantitative analysis, FWHM and Kurtosis could indicate a difference between TA and SOL.

FWHM and Kurtosis have been used as qsi parameters for evaluating neural demyelination and assessing the histological characteristics of carcinomas [23, 27, 28]. FWHM reflects the space in which water molecules can move [17, 18]. Thus, FWHM might represent water molecule diffusion restricted by the cell membrane. Our results showed that FWHM could reflect the difference in cell size between TA and SOL. Kurtosis, which describes the deviation of the water diffusion pattern within a voxel from Gaussian distribution, is considered to reflect changes in microstructural complexity [40]. Our study found that Kurtosis was as effective as FWHM for muscle cell differentiation.

With regard to DWI, in restricted diffusion measurement, it is important to set conditions that sufficiently reflect the organization structure. According to the diffusion equation based on mean square displacement, the diffusion of water molecules is represented as <x^2^> = 2Dt, where D is the diffusion coefficient and t is the diffusion time. From this formula, it can be seen that a sufficient diffusion time is needed to reflect the restricted structure exponentially as the cellular tissue becomes larger. Additionally, because the skeletal muscle cell diameter is larger than the central nervous system cell diameter [41, 42], a diffusion time setting different from that of the nervous system is mandatory [33, 43]. However, during DWI of the skeletal muscles, a short diffusion time similar to that for the central nervous system is often used [10–15]. As the skeletal muscles have short T2 values and a long TE cannot be set, it is difficult to utilize a diffusion time suitable for the skeletal muscles from the viewpoint of SNR with the pulsed-field gradient spin-echo method. However, with the PGSTE method, it is possible to acquire a short TE, long diffusion time, and sufficient SNR for qsi.

In this study, restricted diffusion measurement using qsi was performed. We achieved a high b-value by setting the diffusion time to 200 ms for the PGSTE method. This approach allowed better and more accurate diffusion MRI data acquisition than conventional diffusion acquisition for skeletal muscle, and we successfully evaluated the restricted structure of skeletal muscle cells.

The present study has several limitations. First, our approach did not directly clarify the difference between slow and fast muscle fibers owing to MHC differences. qsi indirectly expressed the differences in muscle fibers according to cell size differences. At present, no imaging technique can directly label MHC. Peng et al. [8] focused on the fact that slow muscle fibers have more collagen than fast muscle fibers [44]. These authors attempted to visualize differences between TA and SOL using T1ρ MRI, which reflects collagen content. This method is also an indirect method similar to qsi. Slow and fast muscle fibers differ in terms of energy metabolism [45, 46], number of mitochondria [47], amount of myoglobin, Ca^2+^ ATPase type [48], and expression of aquaporin 4 membrane protein (AQP4) [49] in addition to cell size and collagen content. It will be necessary to combine MRI techniques focusing on these differences in the future to distinguish muscle quality more completely. Second, qsi involves a long acquisition time. A short acquisition time is required for clinical use. We have successfully performed qsi in a clinical setting within an acceptable acquisition time (<8 min) with reduction of the number of b-value steps and without loss of the characteristic PDF curve and have accordingly performed a clinical operation [23, 26]. The issue of acquisition time may be resolved in clinical trials of qsi for human muscles. Third, there are several other potential confounding factors affecting qsi parameters, such as pennation angle. Because TA and SOL have different fiber angles relative to the plane of the images, this could be a confounding variable while determining the cross-sectional size of the muscle fibers. Studies to resolve this issue are required in the future. Fourth, for both the difference between the two muscles and SD, MRI findings tended to be smaller than histological findings, resulting in divergence between image findings and histological findings. There are two reasons why this phenomenon occurs. The first reason is that qsi does not reflect cell size alone. The diffusion of water molecules is restricted by the cell membrane as a barrier. By measuring the extent of this restricted diffusion to use qsi, we can measure the cell diameter comprising the density of the cell membrane. However, some water molecules penetrate the cell membranes via AQP4. Because the influence of the movement of these water molecules via AQP4 prevents accurate measurement of qsi, the difference in value of qsi is smaller than that of IHC measurement. The second reason is that as a result of the partial volume effect, the numerical values in one voxel are averaged. As a result, variance is suppressed in MRI, and difference in value and SD tend to be small. Even with these effects in mind, muscle discrimination was possible with qsi.

In this study, we focused on the calf that is a part where the distribution of the slow and fast muscle fibers has been already identified. In the future, qsi may be useful for more detailed subtype discrimination, such as differentiation between type IIa and b).

Although the rate of slow and fast muscle fibers is determined to some extent by the genotype [50], the phenotype changes with subsequent training [4] and disuse muscle atrophy [6]. Therefore, qsi in the future may become a method to noninvasively observe phenotype. We believe that qsi has potential as a powerful application not only for medicine but also for sports healthcare, such as distribution measurement of fast and slow muscle fibres, judgment of degree of muscle atrophy, evaluation of training effect, and others.

In conclusion, our findings suggest that qsi is feasible and effective for distinguishing between TA and SOL in an *in vivo* animal model. This technique is a promising method to noninvasively estimate the fiber type ratio in skeletal muscles, and it can be further developed as an indicator of muscle characteristics. This method can help visualize the quality of muscles that could not be visualized until now and will have a considerable influence on the development of sports medicine and clarification of the pathology of several neuromuscular diseases.
